# Robotic versus Laparoscopic Liver Resections for Colorectal Metastases: A Systematic Review and Meta-Analysis

**DOI:** 10.3390/cancers16081596

**Published:** 2024-04-22

**Authors:** Kamil Safiejko, Michal Pedziwiatr, Michal Pruc, Radoslaw Tarkowski, Marcin Juchimiuk, Marian Domurat, Jacek Smereka, Khikmat Anvarov, Przemyslaw Sielicki, Krzysztof Kurek, Lukasz Szarpak

**Affiliations:** 1 Colorectal Cancer Unit, Maria Sklodowska-Curie Bialystok Oncology Center, 15-027 Bialystok, Poland; ksafiejko@onkologia.bialystok.pl (K.S.);; 2 2nd Department of General Surgery, Jagiellonian University Medical College, 31-008 Kraków, Poland; 3 Department of Clinical Research and Development, LUXMED Group, 02-676 Warsaw, Poland; 4 Department of Public Health, International European University, 03-187 Kyiv, Ukraine; 5 Department of Surgical Oncology, Regional Specialist Hospital, 55-220 Legnica, Poland; 6 Laboratory for Experimental Medicine and Innovative Technologies, Department of Emergency Medical Service, Wroclaw Medical University, 51-616 Wroclaw, Poland; 7 Republican Research Center of Emergency Medicine, Ministry of Health of the Republic of Uzbekistan, Tashkent 100107, Uzbekistan; uzkhik@mail.ru; 8 Institute of Outcomes Research, Maria Sklodowska-Curie Medical Academy, 02-315 Warsaw, Poland; 9 Henry JN Taub Department of Emergency Medicine, Baylor College of Medicine, Houston, TX 77030, USA

**Keywords:** colorectal liver metastasis, liver cancer, robotic-assisted surgery, laparoscopic surgery, outcome

## Abstract

**Simple Summary:**

Colorectal cancer can be associated with liver metastasis and may be treated by minimal liver surgery using laparoscopic approaches and robotic surgery. Robotic surgery is of significant use in colorectal surgery and urology. However, there is still no long-term evidence concerning overall survival, and the number of patients operated on using this method remains small. Given the numerous benefits of robotic surgery and the concomitant small number of studies, we conducted a meta-analysis of the operative and short-term oncologic outcomes of laparoscopic versus robotic-assisted liver resection for colorectal liver metastases. The results of the meta-analysis show small differences in blood loss and conversion to open laparotomy rates in favor of robotic surgery. There were no differences in 30-day mortality, and there were also no differences in 1-year, 2-year, or 3-year mortality. The results indicate that both surgical methods are comparable in efficacy and safety.

**Abstract:**

Colorectal cancer is the third most common cancer worldwide, and the liver is the most common localization of metastatic disease. The incidence of minimally invasive liver surgery is increasing, and robotic surgery (RLR) is believed to overcome some limitations of a laparoscopic approach (LRL). We performed a systematic review and meta-analysis of operative and short-term oncologic outcomes of the laparoscopic versus robotic-assisted liver resection for colorectal liver metastases. An online search of PubMed, Embase, Scopus, and the Cochrane databases was performed. Eight studies involving 3210 patients were considered eligible for the meta-analysis. In the LRL group, a higher conversion to open rate (12.4%) was observed compared to the RLR (6.7%; *p* = <0.001). 30-day mortality was 0.7% for the LRL group compared to 0.5% for the RLR group (*p* = 0.76). Mortality in longer periods among LLR and RLR amounted to 18.2% vs. 8.0% for 1-year mortality (*p* = 0.07), 34.1% vs. 26.7% for 2-year mortality (*p* = 0.13), and 52.3% vs. 48.3% for 3-year mortality (*p* = 0.46). The length of hospital stay was 5.6 ± 2.5 vs. 5.8 ± 2.1 days, respectively (*p* = 0.47). There were no significant differences between the incidence of individual complications in the LRL and RLR groups (*p* = 0.78). Laparoscopic or robotic approaches for colorectal liver metastases are comparable in terms of safety and effectiveness. There are significant advantages to robotic surgery, although there is still no long-term evidence concerning overall survival, and the number of patients operated on using RLR remains small.

## 1. Introduction

According to World Cancer Research Fund International, colorectal cancer is the third most common cancer worldwide. It is the third most common cancer in men and the second most common cancer in women [1,2]. The liver is the most common localization of metastatic disease. 20% of patients present synchronous liver metastases, whereas 40% develop metastatic disease at the entire site [3].

Surgery remains the mainstay of treatment for patients with colorectal metastases to the liver, and the incidence of the minimally invasive approach (MILS, Minimal Invasive Liver Surgery) has been increasing [4]. A randomized trial published recently showed the safety of laparoscopic liver resection through overall survival (OS) [5].

While overall survival appears to be equal for both open surgery and laparoscopic approaches, the letter is associated with less morbidity, a shorter hospital stay, and fewer blood transfusions as a result of lower intraoperative blood loss and higher R0 resection rates. It is important to note that a long time of surgery for MILS was pointed out in a cited meta-analysis published by Xie et al. [6]. Robotic surgery is believed to overcome some limitations of a laparoscopic approach. Better binocular vision with a three-dimensional view, wristed tools with better maneuverability, improved instrument dexterity, a lack of tremor, and better depth perception with wristed tools. All of the abovementioned attributes can improve surgical accuracy and reduce surgeon fatigue, particularly during extremely high-demanding procedures such as highly complex liver resections [7].

Laparoscopic liver surgery is used in many centers, and the advantages of this method over open laparotomy are well-known; however, while laparoscopic liver surgery has had an established position in surgical armentaria, experience in the robotic approach to liver surgery still needs to be improved [8]. Although there has been no randomized trial comparing both methods published yet, existing evidence allowed us to perform the presented analysis.

Although reports about the robotic approach to liver surgery are not quite new [9,10] RLR has not gained popularity, perhaps due to the high costs of the procedure. However, despite financial counterarguments, robotic surgery has found a place in colorectal surgery and urology. Together with the advantages mentioned above, this particular approach is also worth considering in the field of hepatobiliary surgery.

We now have substantial meta-analyses on the comparative results of robotic and laparoscopic hepatectomy that show that the outcome of operation time is relevant and robotics leads to longer operation time. There were no significant differences in blood transfusion rate, blood loss, conversion rate, length of hospital stay, or frequency of reoperation between the two groups- robotic and laparoscopic. However, none of these meta-analyses explicitly mentioned colorectal cancer hepatic metastases [11].

The advantages of robotic-assisted surgery over laparoscopic surgery for colorectal liver metastases are still not fully known. As a result, the purpose of this meta-analysis was to compare the operative and short-term oncologic outcomes of laparoscopic vs. robotic-assisted liver resection for colorectal liver metastases.

## 2. Materials and Methods

This systematic review and meta-analysis were performed in accordance with the Reporting Items for Systematic Reviews and Meta-Analyses (PRISMA) statement [12] and registered in the PROSPERO international prospective register of systematic reviews (no. CRD42022376768). The protocol was developed a priori and accepted by all authors, and no protocol changes were made during the study. Due to the character of this study, the ethics committee was not applicable.

### 2.1. Literature Search and Selection

For data collection, we systematically searched PubMed, Embase, Scopus, and the Cochrane Library databases through December 2023, using the terms “robotic” or “robotic-assisted” AND “laparoscopic” AND “liver cancer” OR “liver metastases” AND “colorectal cancer” OR “colorectal liver metastasis surgery”. We have performed searches using keywords (present in the title or abstract), combinations, and limits (humans, adults, and the English language). We have created a reference list, which we then screened. The search strategy was independently peer-reviewed by K.S. and M.P. If necessary, consensus-building or consultation with a third reviewer (L.S.) resolved all differences. Initial search results were merged and imported into the reference management software EndNote^®^ X7. We removed duplicates, and then the remaining items were evaluated for inclusion in the analysis separately by two independent reviewers (K.S. and M.P.) with the use of an open-source citation screening program called ‘abstrackr’ [13]. Any doubts and disagreements were resolved by consensus. The third researcher analyzed the list of relevant articles (L.S.).

We selected studies following our pre-specified clinical research question and the PICOS methodology: (1) Population: adult patients who were diagnosed with colorectal liver metastases and were treated with liver cancer surgery; (2) Intervention: robotic-assisted liver cancer surgery; (3) Comparison: laparoscopic liver cancer surgery; (4) Outcomes, information for survival, mortality, or morbidity; (5) Study design: randomized controlled trials (RCT) or non-RCT comparing robotic-assisted vs. standard laparoscopic surgery for colorectal liver metastases resection. Studies were excluded if: (1) they do not present a comparator group; (2) the literature is conference papers, editorials, reviews, letters, or duplicated publications.

### 2.2. Data Extraction and Quality Assessment

Two researchers (K.S. and L.S.) independently and separately extracted all the following information: first author name, year of publication, region of a cohort, patient characteristics (including the number of patients, age, and sex), intraoperative data (including the type of resection, Pringle maneuver, intraoperative blood loss, intraoperative transfusion, conversion to open laparotomy, operative time), pathological tumor data (including a number of metastases and the size of the largest metastases), or postoperative outcomes (survival rate, disease-free survival rate, length of hospital stay; adverse event types). Any concerns were agreed upon through discussion and analysis with a third researcher (J.S.). The acquired data were entered into a Microsoft Excel spreadsheet (Microsoft Corporation, Redmond, WA, USA) using a specially prepared form. In the absence of data on primary outcomes, we intended to contact the corresponding author of the original study.

We made comparisons of data regarding studies, results, study methodology, and design strengths and weaknesses. In each case, we evaluated the risk of bias using the Rob2 tool for randomized [14] trials and the ROBINS-I bias assessment tool for non-randomized studies [15]. The Rob2 tool includes the following criteria: randomization process, deviations from intended intervention, missing outcome data, measurement of the outcome, and selection of the reported result. In turn, the ROBINS-I tool covered the following criteria: confounding, selection of participants, classification of interventions, deviations from intended interventions, missing data, measurement of outcomes, and selection of the reported result. To visualize the risk of bias assessments, we used the RobVis application [16].

### 2.3. Outcomes

The study’s primary outcome was mortality outcomes, including in different follow-ups (from intraoperative to 3-year follow-up mortality). Other outcomes included length of hospital stay and morbidity occurrence.

### 2.4. Statistical Analysis

Statistical analyses were performed using the Review Manager software (version 5.4, Nordic Cochrane Centre, Cochrane Collaboration) and Stata (version 14, StataCorp, College Station, TX, USA). All statistical tests were two-sided, and the significance level was defined as *p* < 0.05. We have used odds ratios (OR) as the effect measure with 95% confidence intervals (CIs) for dichotomous data and mean differences (MD) with 95% CI for continuous data. In this case, the continuous outcome was reported in a study as median, range, and interquartile range. We estimated means and standard deviations using the formula described by Hozo et al. [17]. The random-effects model was used for all analyses. Heterogeneity was assessed statistically using I^2^ statistics. A low degree of heterogeneity was defined by an I^2^ statistic value < 25%, a moderate degree by an I^2^ statistic value of 25–50%, and a high degree by an I^2^ statistic value > 50% [18]. We have used Egger’s test and funnel plots to check for potential bias and performed funnel plot tests for asymmetry to assess potential publication bias if there were more than ten trials in a single meta-analysis.

## 3. Results

### 3.1. Eligible Studies and Study Characteristics

**Figure 1:**
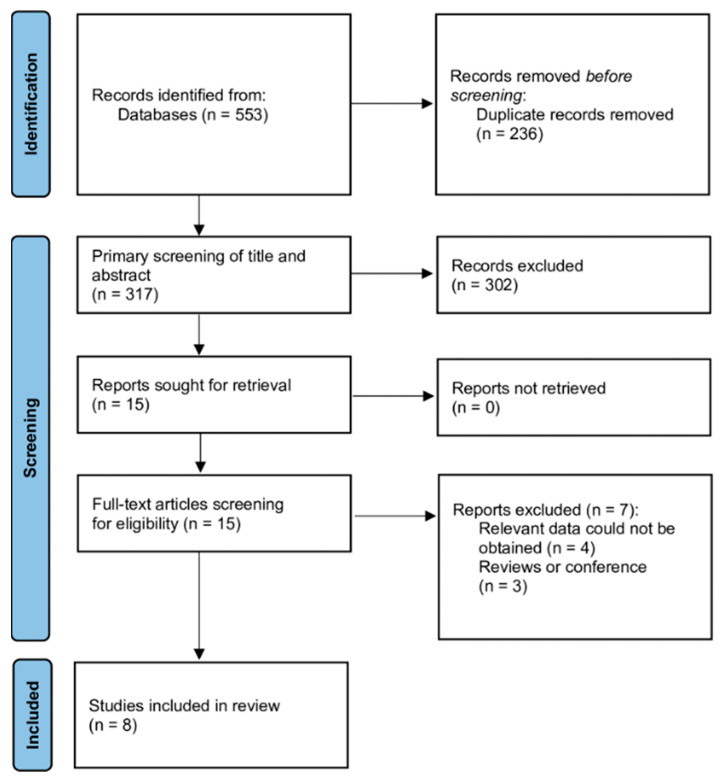
Flow diagram of the search strategy and study selection.

The study selection process is outlined in Figure 1. 533 records were identified in electronic databases. After duplicate removal, 317 records’ titles and abstracts were screened by applying the inclusion and exclusion criteria described previously in the methods section. Fifteen potentially eligible articles were assessed for full-text evaluation. The final analysis included eight trials with a total number of 3210 patients [4,19,20,21,22,23,24,25], of whom 2680 underwent LRL and 530 underwent RLR surgery. Among those studies, one was designed as a prospective study, and seven were retrospective trials. The articles analyzed in this meta-analysis were published between 2020 and 2023. Of the eight trials, two were performed in Italy [22,23], one in China [21], one in the USA [24], and one in Germany [4]. Three studies were international trials [19,20,25]. The characteristics of the included studies appear in Table 1. All the included trials had a low risk of bias. The quality of the included trials is outlined in Supplemental Figures S1–S3.

### 3.2. Patient Characteristics

The detailed characteristics of the patients are presented in Table 2. The mean age of patients in the LLR and RLR groups was 63.5 ± 11.3 and 60.9 ± 10.6 years, respectively (MD = 1.21; 95%CI: 0.39 to 2.02; *p* = 0.004). Males predominated in both groups, with 59.4% in the LLR group and 60.9% in the RLR group, respectively (*p* = 0.49). There was no difference between LLR and RLR in prior abdominal surgery (70.6% vs. 64.9%; *p* = 0.80), prior chemotherapy (59.5% vs. 53.5%; *p* = 0.85), or liver cirrhosis (8.0% vs. 12.9%; *p* = 0.54).

### 3.3. Intraoperative Period Characteristics

The detailed characteristics of the data concerning the intraoperative period and tumor characteristics are presented in Table 3. Pooled analysis showed that the Pringle maneuver was performed statistically significantly more often in the LLR group compared to the RLR group (50.8% vs. 30.6%, respectively; OR = 3.33; 95%CI: 1.53 to 7.22; *p* = 0.002). Six studies reported intraoperative blood loss among the LRL and RRL groups. The pooled analysis showed that blood loss was higher in the LRL group compared to RRL (294.3 ± 312.0 vs. 190.8 ± 118.7 mL, respectively; MD = 178.68; 95%CI: 101.82 to 255.53; *p* < 0.001). More patients in the LRL group than the RLR group needed intraoperative transfusion (30.0% vs. 9.6% respectively; OR = 2.29; 95%CI: 0.79 to 6.63; *p* = 0.13). In the case of LRL, a statistically significantly higher conversion to open rate (12.4%) was observed compared to the RLR (6.7%; OR = 2.18; 95%CI: 1.46 to 3.24; *p* < 0.001).

### 3.4. Outcomes Evaluation

30-day mortality was reported in three trials and was 0.7% for the LRL group compared to 0.5% for the RLR group (*p* = 0.76), while 90-day mortality was 1.3% vs. 0.0% respectively (*p* = 0.58, Supplementary Figure S4). Pooled analysis of 1-year mortality among LRL and RLR groups amounted to 18.2% vs. 8.0%, respectively (*p* = 0.07); in longer follow-up periods, significance disproportion was also not present: 2-year mortality (34.1% vs. 26.7%; *p* = 0.13); 3-year mortality (52.3% vs. 48.3%; *p* = 0.46). Length of hospital stay among LRL and RLR groups varied and amounted to 5.6 ± 2.5 vs. 5.8 ± 2.1 days, respectively (MD = 0.34; 95%CI: −0.59 to 1.28; *p* = 0.47). Table 4 presents a summary of the analyzed 30-day complications. There were no significant differences between the incidence of individual complications in the LRL and RLR groups (*p* > 0.05 for all complications).

## 4. Discussion

Our meta-analysis included 3210 patients with colorectal metastases to the liver treated with surgery, either with a laparoscopic or robotic approach. Analyzed studies described MILS outcomes in patients with colorectal cancer metastases; we have not analyzed those describing primary hepatocellular carcinoma. This remark may be important while keeping in mind the results of previous abdominal surgery in the majority of patients with colorectal cancer (i.e., adhesions, previous chemotherapy, or additional synchronic procedures performed simultaneously at the bowel), which are absent in people with other non-metastatic malignancies localized in the liver.

Age was comparable in both groups, 63.5 ± 11.3 in the laparoscopic (LRL) group and 60.9 ± 10.6 in the robotic (RLR) group; males were the majority in both groups (59.4% and 60.9%, *p* = 0.49). There was no difference between LLR and RLR in prior abdominal surgery (70.6% vs. 64.9%; *p* = 0.80), prior chemotherapy (59.5% vs. 53.5%; *p* = 0.85), or liver cirrhosis (8.0% vs. 12.9%; *p* = 0.54). The LRL group’s tumor size was more extensive: 4.92 ± 2.41 vs. 4.24 ± 1.8 cm (*p* = 0.008). 19.4% of the LRL underwent major liver resections compared to 16.2% in RLR (*p* = 0.008). Intraoperative blood loss was bigger in the laparoscopic group, 294.3 ± 312.0 mL, compared to 190.8 ± 118.7 mL in the robotic one, and intraoperative blood transfusions were noticeably more often in the first group (30.0% vs.’ 9.6%, respectively; *p* < 0.001). Although the RLR group was associated with a higher transfusion rate in the postoperative period (11.7 vs. 6%, *p* = 0.05), the laparoscopic group received a higher number of packed red blood cells in a study published by Masetti [22]. Pringle maneuver was performed more often in LRL (881/1735 (50.8%)) than in the RLR group (78/255 (30.6%)). Gumbs points out that although this maneuver, allowing control of bleeding, was used significantly more during open and laparoscopic approaches compared to the robotic one, after matching, the difference was no longer statistically significant [8]. The higher blood loss in LRL compared to the open surgery study indicates a higher cirrhosis rate in the minimally invasive arm [20]. Before matching, the Pringle maneuver was used significantly more during open and LRL when compared to RLR; after matching, this difference was no longer statistically significant. The observation that patients had, on average, 540 mL more blood loss after LLR and RLR after matching is probably due to the fact that more patients tended to have cirrhosis in the LLR arm.

Operation time was comparable in both groups, 272.9 ± 97.4 and 247.9 ± 81.5 min, respectively (*p* < 0.001). Interesting RLR group characteristics provided a study of Beard: although more patients from this group underwent concomitant one-stage colon or rectal resections or amputations (62.6 vs. 35.2, *p* < 0.001), besides liver surgery, operation time remained equal [19].

The length of hospital stay among LRL and RLR varied and amounted to 5.6 ± 2.5 and 5.8 ± 2.1 days (*p* = 0.47). Conversions to open surgery were more often with the laparoscopic approach (12.4% vs. 6.7%). Lack of positive resection margins (R1) was found more often in the laparoscopic group (22.5%) than in the robotic group (17.3%). Masetti et al. [22], who analyzed the database of 1030 patients from the Italian Group of Minimally Invasive Surgery, reported a greater distance between the tumor and the surgical margin (8 mm [1–10] vs. 3 mm [0–10], *p* < 0.001) in the robotic group. The robotic approach enabled microscopic radicality (*p* = 0.046), while the posterosuperior location of tumors within the liver (*p* < 0.001) and pathological liver parenchyma (*p* < 0.001) were associated with R1 resections more often in the Italian group [22].

There was no intraoperative mortality in both groups. The incidence of total postoperative complications was comparable at 22.4% and 21.9%, respectively, *p* = 0.78. Table 4 shows the details. It is worth noting that the incidence of the biliary fistula was observed at 2.5% in a laparoscopic group, with no cases reported in the robotic one, and bowel complications were present at 3.6% in the LCC group, indicating a lack of the problem shown in the RLR. On the contrary, one complication occurred more often in the RLR group than in the LRL group—a pleural effusion (4.3% vs. 2.8%, respectively; *p* = 0.23). Li et al., reporting the lower rate of complications in the RLR group in their study, explain the difference as an effect of a wider field of view, better positioning and surgical completion, and, thus, more precise operation [21].

There is also a financial aspect to the robotic approach, which is higher than other options. However, looking at Beard’s findings concerning the same operation time at synchronic bowel and liver resection mentioned above [19] and better microscopic radicality, RLR seems to be worth considering.

Conclusions published by Beard et al. based on an analysis of results coming from six specialized, high-patient volume centers show the safety of the robotic approach. The authors indicate two conditions necessary for effectiveness and safety: experience with specialized hepatobiliary surgeons and previous experience with conventional laparoscopy [19]. Robotic liver surgery, as emphasized in the literature, provides substantial improvements compared to traditional minimally invasive liver surgery (MILS), particularly in the treatment of liver metastases situated in the posterior portions. The benefits of using this technique include improved manual skill, accuracy, and the ability to visualize three-dimensional structures. These advantages are crucial for successfully navigating the intricate anatomy of the posterior liver segments. The enhanced 3D visualization, combined with the surgical robot’s capability to suppress tremors and convert the surgeon’s motions into accurate manipulations of the surgical tools, significantly aids in the removal of metastases in these difficult areas. Moreover, research indicates that robotic liver surgery is not only secure but also provides advantages in significant liver resections in terms of surgical duration, blood loss, and hospitalization period compared to traditional laparoscopy. Hence, incorporating robotic treatments into the range of MILS expands the range of conditions that can be treated, providing a valuable tool in the multidisciplinary treatment of liver metastases, particularly those located in less easily reached posterior parts.

Study limitations. The robotic approach to liver metastases is relatively new, and the presented experience comes from highly experienced centers [8]. Furthermore, we have very limited scientific data in the form of only eight studies involving a relatively small number of patients. The published evidence is scarce, and the number of RLR cases is still smaller than those operated on using a laparoscopic approach. Moreover, the retrospective nature of the investigations, with only one randomized trial, restricts our ability to draw highly reliable conclusions. Another limitation of the study was the lack of information regarding the operators’ experience in laparoscopic and robotic surgery, which can affect, among other, the length of a hospital stay or the risk of potential complications. We were also unable to provide the outcomes of the kind of hepatectomy, which also prevented us from declaring precise statistics or referring to the duration of the surgery or other outcomes.

## 5. Conclusions

Laparoscopic or robotic approaches for colorectal liver metastases are comparable in terms of safety and effectiveness. There are some significant advantages to robotic surgery, although there is still no long-term evidence concerning overall survival, and the number of patients operated on using RLR remains small. Larger randomized controlled trials comparing both methods are needed.

## Figures and Tables

**Table 1:** Characteristics of included trials.

Study	Country	Study Design	LLR Group			RLR Group		
			No. of Patients	Age	Sex, Male	No. of Patients	Age	Sex, Male
Balzano et al., 2023 [23]	Italy	Prospective	192	66 ± 12	110 (57.3%)	77	66 ± 12.1	49 (63.6%)
Beard et al. 2020 [19]	Multi-country	Retrospective	514	63 ± 12	314 (61.2%)	115	61 ±11	39 (33.9%)
Cheung et al., 2023 [24]	Multi-country	Retrospective	219	55 ± 4.3	105	73	53.5 ± 4.3	34
Gumbs et al., 2022 [20]	Multi-country	Retrospective	462	63.8 ± 11.7	259 (56.1%)	36	61.8 ± 11.0	21 (58.3%)
Li et al., 2022 [21]	China	Randomized	61	57.51 ± 6.27	38 (62.30%)	61	57.13 ± 586	44 (72.13%)
Masetti et al., 2022 [22]	Italy	Retrospective	953	65.6 ± 10.9	589 (62.7%)	77	65.0 ± 10.6	50 (64.9%)
Radomski et al., 2023 [25]	USA	Retrospective	266	52	NS	79	63	NS
Rahimli et al., 2020 [4]	Germany	Retrospective	13	62.1 ± 12.6	10 (76.9%)	12	63.5 ± 11.3	6 (50.0%)

Legend: LLR: laparoscopic liver resection; RLR: robotic-assisted liver resection; NS: not specified.

**Table 2:** Baseline patient characteristics among included trials.

Outcome	No. of Studies	Event/Participants or Mean ± SD		Events		Heterogeneity between Trials		*p*-Value for Differences across Groups
		LLR	RLR	OR or MD	95%CI	*p*-Value	I^2^ Statistics	
Sex, male	7	1383/2330 (59.4%)	270/443 (60.9%)	0.92	0.74 to 1.15	0.22	28%	0.49
Age, years	7	63.5 ± 11.3	60.9 ± 10.6	1.21	0.39 to 2.02	0.87	0%	0.004
BMI	6	25.97 ± 4.37	26.11 ± 5.66	−0.49	−1.11 to 0.13	0.04	58%	0.12
Prior abdominal surgery	6	1602/2269 (70.6%)	248/382 (64.9%)	0.97	0.74 to 1.26	0.36	9%	0.80
Prior chemotherapy	4	575/966 (59.5%)	115/215 (53.5%)	0.97	0.70 to 1.34	0.82	0%	0.85
Liver cirrhosis	2	48/597 (8.0%)	13/101 (12.9%)	0.70	0.22 to 2.19	0.12	58%	0.54
Preoperative CEA	2	260.2 ± 162.5	61.62 ± 206.2	73.51	−290.02 to 437.004	0.002	90%	0.69

Legend: LLR: laparoscopic liver resection; RLR: robotic-assisted liver resection; CI: confidence interval; BMI: body mass index; CEA: carcinoembryonic antigen test; MD: mean difference; OR: odds ratio; SD: standard deviation.

**Table 3:** Baseline characteristics of intraoperative parameters among included trials.

Outcome	No. of Studies	Event/Participants or Mean ± SD		Events		Heterogeneity between Trials		*p*-Value for Differences across Groups
		LLR	RLR	OR or MD	95%CI	*p*-Value	I^2^ Statistics	
Major resection	3	261/1344 (19.4%)	19/117 (16.2%)	1.15	0.29 to 4.47	0.008	79%	0.84
Minor resection	3	1078/1344 (80.2%)	98/117 (83.8%)	0.86	0.21 to 3.47	0.006	80%	0.83
Pringle maneuver	4	881/1735 (50.8%)	78/255 (30.6%)	3.33	1.53 to 7.22	0.002	79%	0.002
Tumor size	4	3.94± 2.37	4.01 ± 1.82	0.03	−0.56 to 0.63	0.005	76%	0.91
Intraoperative blood loss (mL)	6	294.3 ± 312.0	190.8 ± 118.7	178.68	101.82 to 255.53	<0.001	99%	<0.001
Intraoperative transfusion	5	568/1891 (30.0%)	34/354 (9.6%)	2.29	0.79 to 6.63	<0.001	82%	0.13
Conversion to open	6	312/2522 (12.4%)	30/449 (6.7%)	2.18	1.46 to 3.24	0.76	0%	<0.001
R1 resection	4	328/1457 (22.5%)	39/225 (17.3%)	1.32	0.70 to 2.46	0.16	42%	0.39
Operative time, min	7	272.9 ± 97.4	247.9 ± 81.5	21.50	−5.28 to 48.28	<0.001	97%	0.12

Legend: LLR: laparoscopic liver resection; RLR: robotic-assisted liver resection; CI: confidence interval; MD: mean difference; OR: odds ratio; SD: standard deviation.

**Table 4:** Polled analysis of outcomes among included trials.

Outcome	No. of Studies	Event/Participants or Mean ± SD		Events		Heterogeneity between Trials		*p*-Value for Differences across Groups
		LLR	RLR	OR or MD	95%CI	*p*-Value	I^2^ Statistics	
Mortality								
Intraoperative	1	0/953 (0.0%)	0/77 (0.0%)	NE	NE	NA	NA	NA
30-day	2	13/1931 (0.7%)	2/372 (0.5%)	1.23	0.32 to 4.83	0.66	0%	0.76
90-day	5	10/789 (1.3%)	0/178 (0.0%)	1.78	0.23 to 14.03	0.35	0%	0.58
1-year	3	32/176 (18.2%)	14/176 (8.0%)	2.56	0.94 to 6.98	0.15	53%	0.07
2-years	2	60/176 (34.1%)	47/176 (26.7%)	1.42	0.90 to 2.24	0.69	0%	0.13
3-years	2	92/176 (52.3%)	85/176 (48.3%)	1.17	0.77 to 1.78	0.56	0%	0.46
Hospital length of stay, days	7	5.6 ± 2.5	5.8 ± 2.1	0.34	−0.59 to 1.28	<0.001	99%	0.47
30-days complications								
Total postoperative complications	7	404/1806 (22.4%)	106/482 (21.9%)	1.04	0.81 to 1.32	0.25	26%	0.78
Major complications	7	167/2123 (7.9%)	28/449 (6.2%)	1.37	0.91 to 2.08	0.31	17%	0.13
Ascites	1	7/953 (0.7%)	0/77 (0.0%)	1.23	0.07 to 21.71	NA	NA	0.89
Haemorrhage	2	19/1014 (1.9%)	0/138 (0.0%)	3.06	0.37 to 25.50	1.00	0%	0.30
Coagulopathy	1	4/953 (0.4%)	0/77 (0.0%)	0.73	0.04 to 13.77	NA	NA	0.84
Biliary fistula	2	25/1014 (2.5%)	0/138 (0.0%)	4.45	0.56 to 35.20	0.89	0%	0.16
Bowel complications	1	34/953 (3.6%)	0/77 (0.0%)	5.82	0.35 to 95.77	NA	NA	0.22
Surgical site infection	1	17/953 (1.8%)	1/77 (1.3%)	1.38	0.18 to 10.51	NA	NA	0.76
Intra-abdominal abscess	2	32/1014 (3.2%)	3/138 (2.2%)	1.39	0.40 to 4.84	0.72	0%	0.60
Pneumonia	1	19/953 (2.0%)	2/77 (2.6%)	0.76	0.17 to 3.34	NA	NA	0.72
Pleural effusion	2	28/1014 (2.8%)	6/138 (4.3%)	0.62	0.15 to 2.55	0.22	34%	0.50
Pneumothorax	1	2/953 (0.2%)	0/77 (0.0%)	0.41	0.02 to 8.56	NA	NA	0.56
Deep vein thrombosis	1	2/953 (0.2%)	0/77 (0.0%)	0.41	0.02 to 8.56	NA	NA	0.56
Pulmonary embolism	1	1/953 (0.1%)	0/77 (0.0%)	0.24	0.01 to 6.04	NA	NA	0.39
Posthepatectomy liver failure	1	5/953 (0.5%)	0/77 (0.0%)	0.90	0.05 to 16.41	NA	NA	0.94
30-d readmission	4	49/598 (8.2%)	16/267 (6.0%)	1.39	0.76 to 2.56	0.46	0%	0.29
30-d reoperation	4	18/600 (3.0%)	4/267 (1.5%)	1.72	0.61 to 4.87	0.57	0%	0.31

Legend: LLR: laparoscopic liver resection; RLR: robotic-assisted liver resection; CI: confidence interval; MD: mean difference; OR: odds ratio; SD: standard deviation; NA: not applicable, NE: not estimable.

## Data Availability

The data that support the findings of this study are available on request from the corresponding author (L.S.).

## Supplementary Materials

The following supporting information can be downloaded at: https://www.mdpi.com/article/10.3390/cancers16081596/s1, Figure S1: A summary table of review authors’ judgments for each risk of bias item for randomized study; Figure S2: A summary table of review authors’ judgments for each risk of bias item for non-randomized trials; Figure S3: A plot of the distribution of review authors’ judgments across nonrandomized studies for each risk of bias item; Figure S4: Forest plot of mortality in different follow-up periods among LLR and RLR groups.
