# Supplementary material for: Robotic versus Laparoscopic Liver Resections for Colorectal Metastases: A Systematic Review and Meta-Analysis
Source: Cancers (Basel). 2024 Apr 22;16(8):1596. doi: 10.3390/cancers16081596 (PMC11048946; doi:10.3390/cancers16081596)
Supplement: Supplementary file 1 [file cancers-16-01596-s001.zip › cancers-2940885-supplementary.pdf]

Robotic versus Laparoscopic Liver Resections for Colorectal Metastases:

A Systematic Review and Meta-Analysis

Supplementary Digital File

CONTENT:

Figure S1. A summary table of review authors' judgements for each risk of bias item for randomized study. 2

Figure S2. A summary table of review authors' judgements for each risk of bias item for non randomized trials. 2

Figure S3: A plot of the distribution of review authors' judgements across non randomized studies for each risk of bias item 3

Figure S4: Forest plot of mortality in different follow-up periods among LLR and RLR groups. The center of each square represents the odds ratios for individual trials, and the corresponding horizontal line stands for a 95% confidence interval. The diamonds represent pooled results. 3

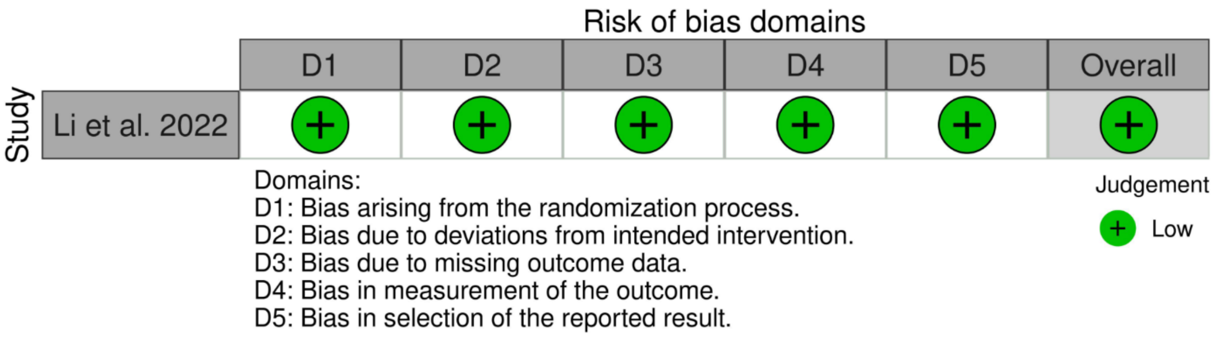

Figure S1. A summary table of review authors' judgements for each risk of bias item for randomized study.

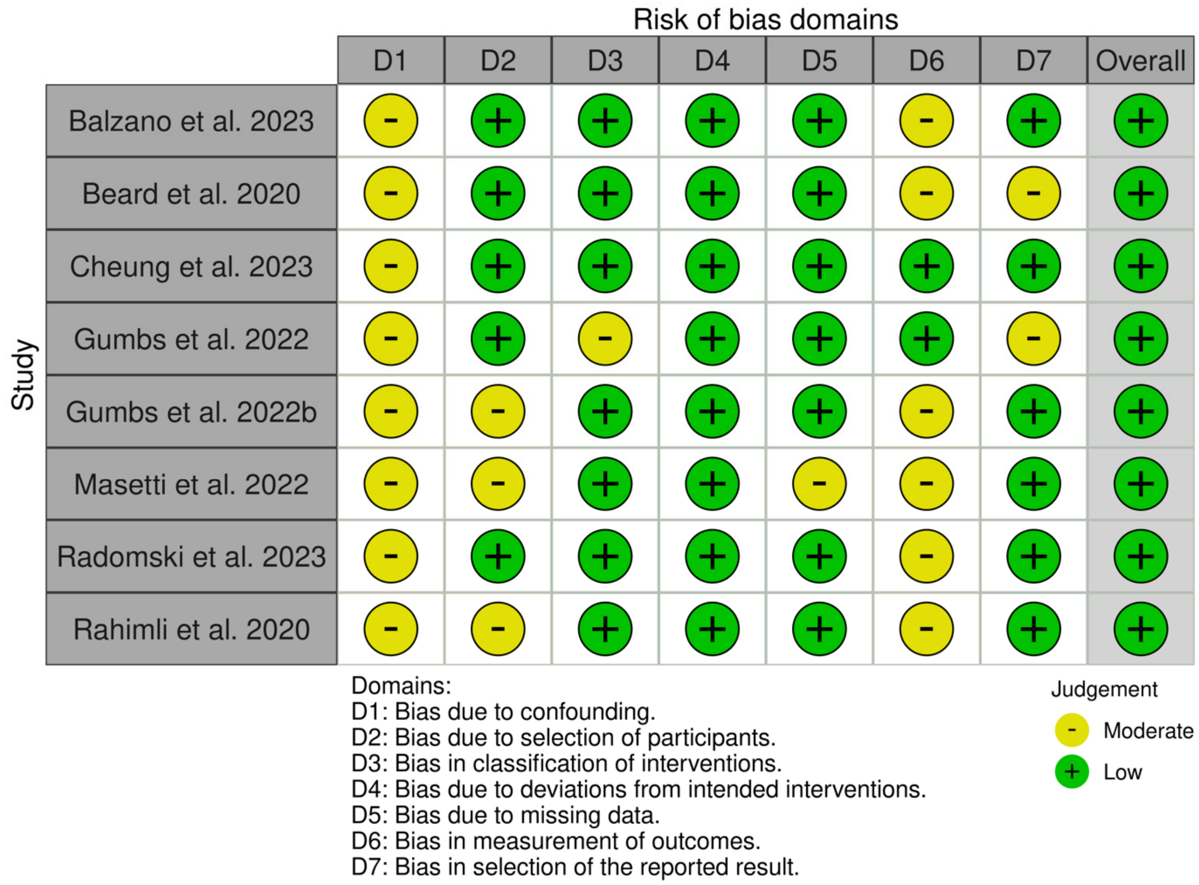

Figure S2. A summary table of review authors' judgements for each risk of bias item for non randomized trials.

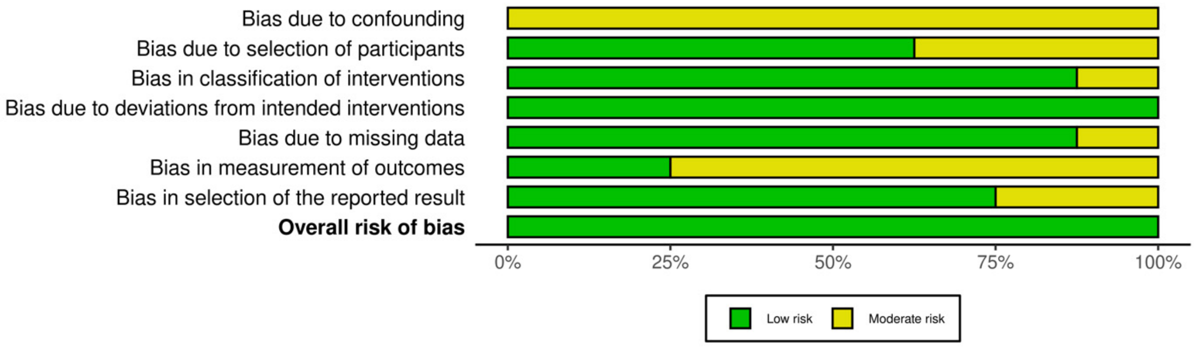

Figure S3. A plot of the distribution of review authors' judgements across non randomized studies for each risk of bias item.

	LLR		RLR			Odds Ratio
**Study or Subgroup**	**Events**	**Total**	**Events**	**Total**	**Weight**	**M-H, Random, 95% CI**
**1.6.2 30-d mortality**						
Cheung 2023	4	219	0	73	21.6%	3.07 [0.16, 57.70]
Radomski 2023	5	266	1	79	39.8%	1.49 [0.17, 12.98]
test	4	1446	1	220	38.6%	0.61 [0.07, 5.46]
**Subtotal (95% CI)**		**1931**		**372**	**100.0%**	**1.23 [0.32, 4.83]**
Total events	13		2			
Heterogeneity: Tau² = 0.00; Chi² = 0.83, df = 2 (P = 0.66); I² = 0%						
Test for overall effect: Z = 0.30 (P = 0.76)						
**1.6.3 90-d mortality**						
Balzano 2023	0	192	0	77		Not estimable
Cheung 2023	6	219	0	73	51.0%	4.48 [0.25, 80.42]
Gumbs 2022	4	378	0	28	49.0%	0.68 [0.04, 13.04]
**Subtotal (95% CI)**		**789**		**178**	**100.0%**	**1.78 [0.23, 14.03]**
Total events	10		0			
Heterogeneity: Tau² = 0.00; Chi² = 0.87, df = 1 (P = 0.35); I² = 0%						
Test for overall effect: Z = 0.55 (P = 0.58)						
**1.6.4 1-yr**						
Beard 2020	19	115	5	115	47.9%	4.35 [1.57, 12.10]
Li 2022	13	61	9	61	52.1%	1.56 [0.61, 3.99]
**Subtotal (95% CI)**		**176**		**176**	**100.0%**	**2.56 [0.94, 6.98]**
Total events	32		14			
Heterogeneity: Tau² = 0.28; Chi² = 2.11, df = 1 (P = 0.15); I² = 53%						
Test for overall effect: Z = 1.83 (P = 0.07)						
**1.6.5 2-yr**						
Beard 2020	39	115	29	115	64.1%	1.52 [0.86, 2.69]
Li 2022	21	61	18	61	35.9%	1.25 [0.58, 2.69]
**Subtotal (95% CI)**		**176**		**176**	**100.0%**	**1.42 [0.90, 2.24]**
Total events	60		47			
Heterogeneity: Tau² = 0.00; Chi² = 0.16, df = 1 (P = 0.69); I² = 0%						
Test for overall effect: Z = 1.50 (P = 0.13)						
**1.6.6 3-yr**						
Beard 2020	57	115	55	115	65.6%	1.07 [0.64, 1.80]
Li 2022	35	61	30	61	34.4%	1.39 [0.68, 2.84]
**Subtotal (95% CI)**		**176**		**176**	**100.0%**	**1.17 [0.77, 1.78]**
Total events	92		85			
Heterogeneity: Tau² = 0.00; Chi² = 0.34, df = 1 (P = 0.56); I² = 0%						
Test for overall effect: Z = 0.75 (P = 0.46)						

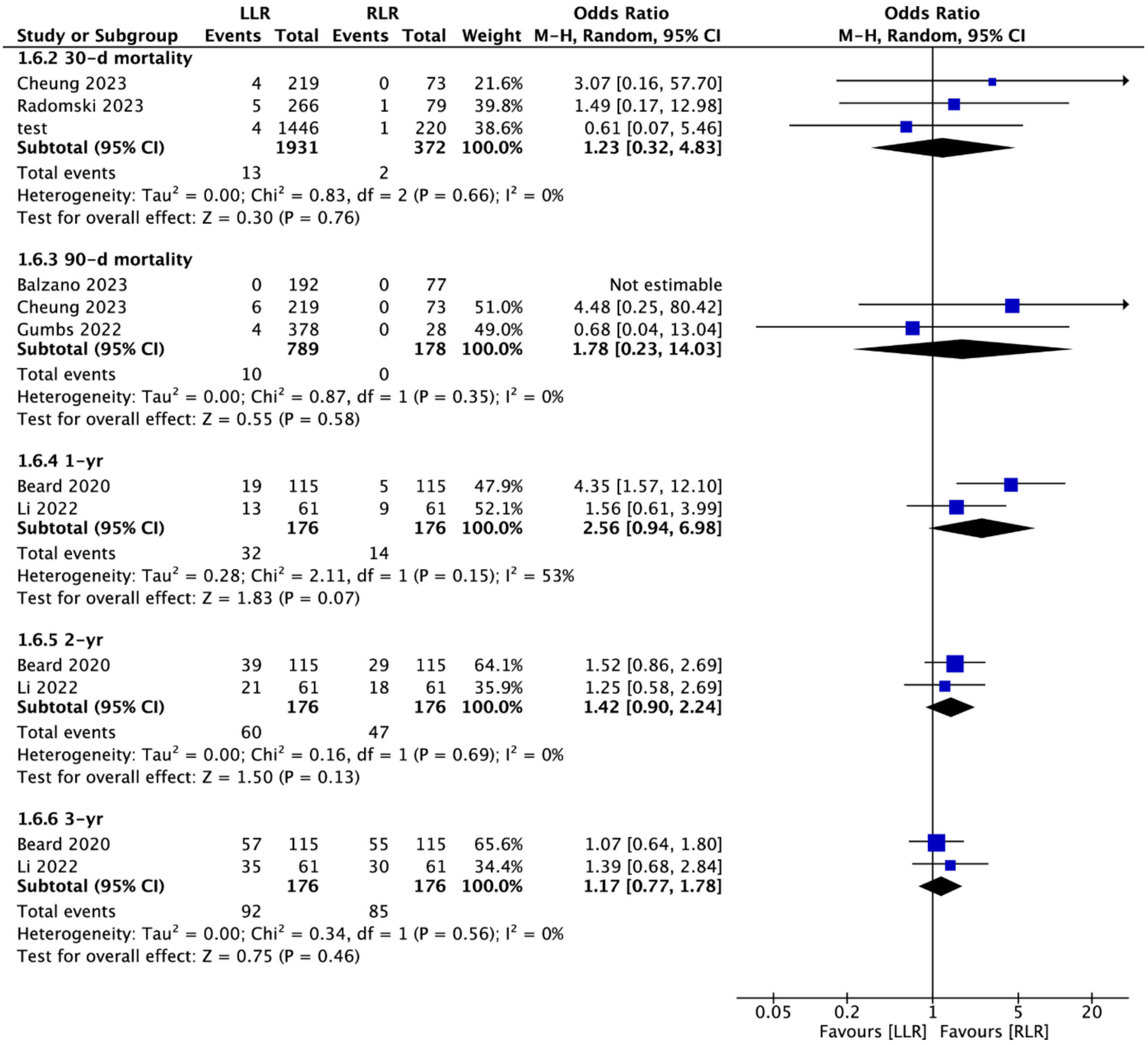

Figure S4. Forest plot of mortality in different follow-up periods among LLR and RLR groups. The center of each square represents the odds ratios for individual trials, and the corresponding horizontal line stands for a 95% confidence interval. The diamonds represent pooled results.
